# The genome of New Zealand trevally (Carangidae: *Pseudocaranx georgianus*) uncovers a XY sex determination locus

**DOI:** 10.1186/s12864-021-08102-2

**Published:** 2021-11-02

**Authors:** Andrew Catanach, Mike Ruigrok, Deepa Bowatte, Marcus Davy, Roy Storey, Noémie Valenza-Troubat, Elena López-Girona, Elena Hilario, Matthew J. Wylie, David Chagné, Maren Wellenreuther

**Affiliations:** 1 The New Zealand Institute for Plant & Food Research Ltd, Christchurch, New Zealand; 2 Department of Bioinformatics, University of Applied Sciences Leiden, Leiden, The Netherlands; 3 The New Zealand Institute for Plant & Food Research Ltd, Nelson, New Zealand; 4 The New Zealand Institute for Plant & Food Research Ltd, Palmerston North, New Zealand; 5 The New Zealand Institute for Plant & Food Research Ltd, Te Puke, New Zealand; 6 The New Zealand Institute for Plant & Food Research Ltd, Auckland, New Zealand; 7 School of Biological Sciences, The University of Auckland, Auckland, New Zealand

**Keywords:** *Pseudocaranx georgianus*, Sex determination, Teleost, Aromatase cyp19a1a, *cyp19a1b*, Cytochrome P450 aromatase, Genomics, Sex system, Carangidae, Molecular sex markers, Aquaculture and assembly

## Abstract

**Background:**

The genetic control of sex determination in teleost species is poorly understood. This is partly because of the diversity of mechanisms that determine sex in this large group of vertebrates, including constitutive genes linked to sex chromosomes, polygenic constitutive mechanisms, environmental factors, hermaphroditism, and unisexuality. Here we use a *de novo* genome assembly of New Zealand silver trevally (*Pseudocaranx georgianus*) together with sex-specific whole genome sequencing data to detect sexually divergent genomic regions, identify candidate genes and develop molecular makers.

**Results:**

*The de novo* assembly of an unsexed trevally (Trevally_v1) resulted in a final assembly of 579.4 Mb in length, with a N50 of 25.2 Mb. Of the assembled scaffolds, 24 were of chromosome scale, ranging from 11 to 31 Mb in length. A total of 28,416 genes were annotated after 12.8 % of the assembly was masked with repetitive elements. Whole genome re-sequencing of 13 wild sexed trevally (seven males and six females) identified two sexually divergent regions located on two scaffolds, including a 6 kb region at the proximal end of chromosome 21. Blast analyses revealed similarity between one region and the aromatase genes *cyp19 (a1a/b)* (E-value < 1.00E-25, identity > 78.8 %). Males contained higher numbers of heterozygous variants in both regions, while females showed regions of very low read-depth, indicative of male-specificity of this genomic region. Molecular markers were developed and subsequently tested on 96 histologically-sexed fish (42 males and 54 females). Three markers amplified in absolute correspondence with sex (positive in males, negative in females).

**Conclusions:**

The higher number of heterozygous variants in males combined with the absence of these regions in females support a XY sex-determination model, indicating that the trevally_v1 genome assembly was developed from a male specimen. This sex system contrasts with the ZW sex-determination model documented in closely related carangid species. Our results indicate a sex-determining function of a *cyp19a1a*-like gene, suggesting the molecular pathway of sex determination is somewhat conserved in this family. The genomic resources developed here will facilitate future comparative work, and enable improved insights into the varied sex determination pathways in teleosts. The sex marker developed in this study will be a valuable resource for aquaculture selective breeding programmes, and for determining sex ratios in wild populations.

**Supplementary Information:**

The online version contains supplementary material available at 10.1186/s12864-021-08102-2.

## Introduction

The genetic basis of sex determination (SD) in animals has long fascinated researchers due to the relationship of this trait with reproduction and Darwinian fitness [1, 2]. Traditionally, sex determination was assumed to be a relatively conserved trait across vertebrates. However, recent research on teleost fishes has shown that this is not the case, and that teleosts display a remarkable diversity in the ways sex is determined. These different mechanisms, which include heterogamety for males (males XY females XX) or heterogamety for females (males ZZ females ZW), multiple sex chromosomes and genes determining sex, environmental influences (temperature-dependent), epigenetic sex determination and hermaphroditism, have each independently originated numerous times [1, 3, 4]. The evolutionary lability of SD, and the corresponding rapid rate of turn-over among different modes, makes the teleost clade an excellent model to test theories regarding the evolution of SD adaptations [5, 6].

Teleosts consist of over 30,000 species, making them the largest group of vertebrates [7]. This diversity in species corresponds to a high phenotypic diversity and associated capacity of adaptation in physiological, morphological and behavioural traits. Reproductive systems vary largely, and strategies range from gonochorism, protandrous, protogynous and simultaneous hermaphroditism [8]. These reproductive strategies emerged independently in different lineages, demonstrating a polyphyletic origin. Looking across fish families and genera, the genetic basis of SD can be profoundly different, and can also be determined entirely by external factors, e.g. social structure or attainment of a critical age [9]. Importantly, it should be noted that for most fish species it is unknown how sex is genetically determined and what the genetic architecture is of sex determination (e.g. monogenic vs. polygenic architecture).

**Fig. 1:**
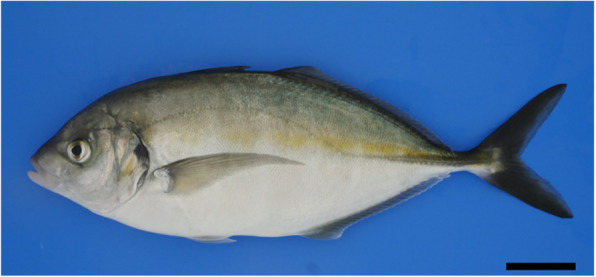
Trevally (Carangidae: *Pseudocaranx georgianus*) individual from the breeding programme at Plant and Food Research in Nelson, New Zealand. Trevally are also known as ‘araara’, its indigenous Māori name in New Zealand. The black scale bar is 5 cm

The New Zealand silver trevally *Pseudocaranx georgianus* (hereafter referred to as trevally, Fig. 1) also known as ‘araara’, its indigenous Māori name, is a teleost fish species of the family Carangidae. This family consists of approximately 30 genera which together contain around 151 species worldwide [10], yet SD has only been studied in a few species of this family. These studies have revealed that all of the carangid species studied to date are gonochoristic and that SD is genetically controlled [8, 11, 12], which indicates that individuals are either a genetic male or female. While this family is morphologically diverse, the number of karyotypes appears mostly conserved, and species are predominantly characterised by 2*n* = 48 acrocentric chromosomes, which is likely the ancestral state [13]. Studies on the sex determination systems in carangids appear to be limited, however, in Japanese amberjack (*Seriola quinqueradiata*) and golden pompano (*Trachinotus oyatus*), ZZ-ZW systems have been documented [14, 15].

Trevally is a pelagic species and abundant in the coastal waters of Oceania, spanning from the coastal regions of the North Island and the top of the South Island of New Zealand to southern Australia [16–18]. The fish grows to a maximum length of 1.2 m and 18 kg, and can reach 25 years [19]. Their bodies are elongated, with the upper portion being bluish-silver, the lower portion of the fish is silver and the sides are yellow silver in colour (see Figs. 1 and [19]). They commonly school with size-similar individuals and forage on plankton and bottom invertebrates [16]. The species is highly sought-after for sashimi in Asia, and several countries are trying to establish aquaculture breeding programmes e.g. [20]. Adults of this species are sexually monomorphic externally, as observed in other carangids [21, 22]. Trevally have a firm musculature around their abdominal cavity, making phenotypic sexing difficult. Thus, sex can typically only be determined subsequent to lethal sampling or by gonopore cannulation to retrieve a gonadal biopsy. This technique, however, can only be applied to broodstock in the advanced stages of gametogenesis shortly before or during the reproductive season and can injure the fish. Sexual maturation takes 3–4 years in captivity, meaning that sex information can only be gathered following that stage. Hence, understanding the genetic basis of SD in trevally would allow the design of molecular markers to facilitate sexing of the individuals early in life and in a less-invasive way.

The overarching goal of this study was to identify the genetic underpinnings of SD in trevally. To achieve this, we (1) *de novo* assembled a reference genome and (2) identified sexually divergent genomic regions based on sequencing depth and variant detection using whole genome re-sequencing of male and female fish. Then, (3) candidate genes for SD were identified and (4) molecular markers were designed and validated using individuals sexed by gonadal histology. We discuss our findings about SD in this species and highlight the resulting applications, and compare them to other teleost species to draw general conclusions about SD in this group.

## Materials and methods

### Broodstock collection and rearing of F_1_ offspring

Trevally samples were collected from a founding (F_0_) wild-caught captivity-acclimated population and a captive-bred (F_1_) generation produced by The New Zealand Institute for Plant and Food Research Limited (PFR) in Nelson, New Zealand. All fish were maintained under ambient photoperiod and water temperatures of filtered flow-through seawater. Fish were fed daily to satiation on a diet consisting of a commercial pellet feed (Skretting and/or Ridely) supplemented with frozen squid (*Nototodarus* spp.) and an in-house mixed seafood diet enriched with vitamins. Details about the fish rearing conditions can be found in Supplementary Material 1: Fish rearing details.

In 2017, a single two-year-old F_1_ juvenile was sampled for the genome assembly (section Genome sequencing and assembly), while five additional fish were sampled to annotate the genome (tissues sampled: skin, white muscle, gill, liver, kidney, brain and heart tissues) (Supplementary Material 2: RNA extraction for transcriptome sequencing). Three-year-old F_1_ individuals (*n*=96) were lethally sexed and sampled in 2018 and used for validation of the sex marker.

### Genome sequencing and assembly

#### Short-insert library preparation, sequencing, and assembly

High-quality DNA for the genome assembly was extracted from heart tissue as described in Supplementary Material 3: DNA extraction. Dovetail Genomics (Scotts Valley, CA, USA) was contracted to conduct the *de novo* sequencing project, which consisted of a short insert library and two long range libraries (Hi-C and Chicago). The Illumina short-insert library was prepared with randomly fragmented DNA according to the manufacturer’s instructions. The library was sequenced on an Illumina HiSeq X platform using paired-end (PE) 150 bp sequencing. The data were trimmed for low-quality bases and adapter contamination using Trimmomatic and Jellyfish [23] with in-house software to profile the short insert reads at a variety of k-mer values (25, 43, 55, 85 and 109) to estimate the genome size, and fit negative binomial models to the data. The resulting profiles suggested a k-mer size of 43 was optimal for assembly. The contigs were assembled into scaffolds using Meraculous [24], with a k-mer size of 43, a minimum k-mer frequency of 12, and the diploid nonredundant haplotigs mode.

### Chicago library preparation and sequencing

Second, following the *de novo* assembly with Meraculous, a Chicago library was prepared according to the methods described in Putnam et al. [25]. Briefly, ∼500 ng of high molecular weight genomic DNA was reconstituted *in vitro* into chromatin and subsequently fixed with formaldehyde. The fixed chromatin was then digested with DpnII, the 5′ overhangs were filled in with biotinylated nucleotides and free blunt ends were ligated. After ligation, crosslinks were reversed and the DNA was purified from any protein. The purified DNA was then treated to remove biotin that was not internal to ligated fragments and the resulting DNA was sheared to ∼350 bp mean fragment size using a Bioruptor Pico. Sequencing libraries were prepared from the sheared DNA using NEBNext Ultra enzymes (New England Biolabs, Inc.) and Illumina-compatible adapters. The biotin-containing fragments were isolated using streptavidin beads before PCR enrichment of each library. The amplified libraries were finally sequenced on an Illumina HiSeq X platform using PE 150 reads to approximately 90X depth.

### Dovetail Hi-C library preparation and sequencing (multiple libraries)

Third, a Dovetail Hi-C library was prepared from the heart tissue preserved in RNAlater following the procedures outlined in Lieberman-Aiden et al. [26]. This library was based on a genome-wide Chromatin Conformation Capture protocol using proximity ligation. Briefly, formaldehyde was used to fix chromatin in place in the nucleus, which was then extracted and digested with DpnII. The 5′ overhangs were filled with biotinylated nucleotides, and free blunt ends were ligated. After ligation, the crosslinks were reversed and the DNA was purified from remaining protein. Biotin that was not internal to ligated fragments was removed from the purified DNA, which was subsequently sheared to ∼350 bp mean fragment size using a Bioruptor Pico. The sequencing libraries were then prepared using NEBNext Ultra enzymes and Illumina-compatible adapters. Before PCR enrichment of the library, biotin-containing fragments were isolated using streptavidin beads. The resulting library was sequenced on an Illumina HiSeq X Platform using PE 150 reads to approximately 60X depth.

### Assembly scaffolding with HiRise

To scaffold and improve the trevally *de novo* assembly, Dovetail staff input the Meraculous assembly, along with the shotgun reads, Chicago library reads, and Dovetail Hi-C library reads into the HiRise pipeline [25] to conduct an iterative analysis. First, the shotgun and Chicago library sequences were aligned to the draft contig assembly using a modified SNAP read mapper (http://snap.cs.berkeley.edu). Second, the separations of Chicago read pairs mapped within draft scaffolds were analysed to produce a likelihood model for genomic distance between read pairs. This model was used to identify and break putative misjoins, score prospective joins, and make joins above a threshold. Finally, after aligning and scaffolding the draft assembly using the Chicago data, the Chicago assembly was aligned and scaffolded using Dovetail Hi-C library sequences following the same method. After scaffolding, the short-insert sequences were used to close remaining gaps between contigs where possible.

### Assembly polishing and contiguity statistics

After receiving the assembly from Dovetail, *de novo* repeats were identified using RepeatModeler v1.0.11 (http://www.repeatmasker.org/RepeatModeler.html) with the NCBI search engine (rmblast version). Repeats were classified by RepeatModeler into simple, tandem and interspersed repeats and masked using RepeatMasker v4.0.5 [27].

### Genome annotation

Automated gene models were predicted using the BRAKER2 pipeline v2.1.0 [28] with trevally RNA sequences and the trevally genome assembly as input. Gene and genome completeness were evaluated using BUSCO v3.0.2 [29] using the vertebrata_odb9 lineage set (containing 2586 genes). Functional annotations were assigned to the gene models using blastx [30] to search for similarities between the translated transcriptome gene-locus models and a peptide database using 88,504 peptide sequences of Zebrafish *Danio rerio* and 39,513 peptide sequences of *Seriola lalandi* (downloaded from NCBI using E-utilities version 11.4, 7th September 2020). The results from these searches were merged with species-specific genome-wide annotation for *Danio rerio* provided in the package org.Dr.eg.db [31], using Entrez stable gene identifiers [32] and Genbank accessions to annotate BLASTX alignments of gene models. Common Gene Locus (gene model g1 . g28000) from blast reports were also used to marry up Zebrafish and Kingfish accession and description information.

### Whole genome sequencing of sexed F_0_ broodstock

Sampling of the 13 remaining broodstock (of the original 21) took place during February 2017. Fin tissue (fin clips) were placed directly into chilled 96 % ethanol, heated to 80 °C for 5 min within 1 h of collection, and then stored at -20 °C. Total genomic DNA was extracted as described in Supplementary Material 3: DNA extraction. High quality DNA was used to create short insert (300 bp) libraries (Illumina) which were sequenced by AGRF (PE reads, 125 bp long).

### Whole genome sequence read alignment and variant detection

FASTQ files of reads belonging to the 13 sexed F_0_ broodstock were quality filtered using Trimmomatic v0.36 [33] with a sliding window size of 4, a quality cut-off of 15 and the minimum read length set at 50. Filtered FASTQ files were aligned to the reference genome Trevally_v1 using BWA-MEM v0.7.17 [34]. Aligned BAM files of two sequencing lanes per individual fish were merged using Samtools v1.7 [35]. Read groups were added and duplicates were removed from merged BAM files using Picard Tools v2.18.7, and sorted and indexed using Samtools. Variant calling was done on the whole cohort of 13 fish using freebayes-parallel v1.1.0 (https://github.com/freebayes).

### Genome-wide detection of sex-linked variants

Two strategies were used for detecting sex-associated regions using the re-sequencing data from the 13 sexed broodstock (Supplementary Fig. 1). First, a read-depth variation approach was employed to detect regions where read-mapping of samples is absent or reduced in one sex, characteristic of mapping of reads of either Y chromosomes in males of XY species, or of reads of W chromosomes of females in ZW species. Second, a variant (SNPs or indels) state (homozygous or heterozygous) approach was employed, searching for regions characteristic of divergence of sex chromosomes (X versus Y, Z versus W). The heterogametic sex is expected to possess high frequencies of variants that are heterozygous in regions that have diverged in the sex chromosome.

Alignments to scaffolds shorter than 3000 bp were excluded from bam files using an in-house BASH script with AWK. To detect regions with variable read-depths between males and females, for each sample, read-depth was calculated per base using Samtools v1.7 [32]. To determine sex-associated variation in read depth, mean depth was calculated for 1 kb bins for each sample, and t-tests (Welch Two Sample t-test, R v3.5.3) were conducted to test differences between means of males and females, for each 1 kb bin. P-values were converted into –log10P values and 1 kb windows with –log10P values greater than 2 were retained for plotting.

To determine association of variant state with sex (heterozygous in one sex, homozygous in the other), a VCF file of all 13 samples was generated using vcftools v0.1.14 [36] and was converted into genotypes using vcfR v.1.8.0 [37] in R v3.5.0. Fisher’s exact test (R v3.5.0) was used to test the association of sex with genotype states (usually 0/0, 0/1 or 1/1). P-values were converted into –log10P values, and variants that had states entirely associated with sex were retained for plotting. Whole genome plotting of regions of significant read depth variation and variant state was done using Circlize [38] and plotting of individual scaffolds was done using ggplot2 [39] in R v3.5.0.

### Identification of candidate genes related to sex determination

Teleost SD candidate genes were identified and compiled from publications from 1998 and onwards using the search terms: sex determination, *Pseudocaranx georgianus*, Carangidae, Perciformes, teleost, and fish in combination with sex determination and sex genes, in Google Scholar (parsed from 1 to 2019 to 1 October 2019). Sequences of candidate genes were downloaded from NCBI and used to query the trevally reference genome Trevally_v1 using blastn v2.2.25 [40], filtering for E-values < 1e-10, and alignment lengths and bit scores greater than 99. Gene models were developed based on sequence similarity at the peptide level using blastx and blastp against similar sequences of teleost fishes of the non-redundant protein database of NCBI [41]. To determine the class of the trevally sex-associated gene and its paralogues, searches of the Protein database at NCBI were made using *cyp19a1a* and *cyp19a1b* as search terms. Selections of ten peptides of each of these genes were used to generate a guide tree along with human *cyp1a1* as an outgroup using the Clustal Omega web server at EMBL-EBI [42].

### Sex phenotyping for marker development

For the development and validation of a molecular sex marker in trevally, gonadal tissues were collected from three-year-old F_1_ individuals (*n*=96). In brief, fish were subjected to complete sedation and euthanasia by overdose in anaesthetic (> 50 ppm AQUI-S®; Aqui-S New Zealand Ltd, Lower Hutt, New Zealand) followed by cervical dislocation with a sharp knife.

A fragment of gonadal tissue was dissected and fixed in a solution of 4 % formaldehyde-1 % glutaraldehyde for at least 48 h at 4 °C. Fixed samples were then dehydrated through an ethanol series before being embedded in paraffin (Paraplast, Leica Biosystems Richmond Inc, Richmond IL, USA). Serial sections cut to a thickness of 5 μm were obtained using a microtome (Leica RM2125RT, Leica Microsystems Nussloch GmbH, Germany) and stained in Gill 2 hematoxylin (Thermo Scientific Kalamazoo, MI, USA) and counterstained with eosin. Histological sections were examined under a light compound microscope (Olympus BX50) for the presence of oocytes or spermatogonia and photographed with a digital camera (Nikon DS-Ri2) to confirm the sex of each individual.

### Sex marker development and validation

Fin clips were collected from the 96 individual F_1_ fish and placed directly into chilled 96 % ethanol, heated to 80 °C for 5 min and then stored in a -20 °C freezer. Total genomic DNA was extracted as described above. Three types of genetic markers were developed in the sex-linked regions. PCR primers were designed using the Primer3 v4.1.0 web application. Y-specific markers were designed using male sequences where there is an absolute absence in females, so that PCR only amplifies the Y allele. Gene-based primers were designed with default parameters using the trevally ortholog of *cyp19a1a* from *Seriola lalandi* (HQ449733.1). PCR primers for High Resolution Melting (HRM) were designed around the sexually divergent SNPs by flanking the SNPs with 100 bp on each side.

HRM markers were screened using PCR conditions and mix described in Guitton et al. [43] using genomic DNA extracted from fin clips of these fish. Y-allele specific and candidate gene-based markers were screened as sequence-characterized amplified regions (SCAR) markers as described in Bus et al. [44]. PCR conditions were first tested on eight individual samples to verify PCR amplification and presence (in males) absence (in females) polymorphism, then screened on the population of 96 sexed fish.

## Results

### Genome sequencing and assembly

In total, 412,758,157 paired-end Illumina short reads were generated from an F_1_ unsexed trevally, of which 97.4 % were retained after trimming. K-mer analysis (k=43) resulted in 0.71 % of heterozygous SNPs and an estimated genome size of 646 Mb. The total input sequencing data pre-assembly was approximately 121 Gb, which is equivalent to 187.3× coverage.

The whole genome assembly yielded 2,006 scaffolds greater than 1 kb, for a total assembly size of 579.4 Mb (89 % of estimated genome) and a N50 (scaffold) of 25.2 Mb. Of this total assembly, 574.8 Mb (99.2 % of the total assembly and 88.8 % of the k-mer estimated genome size) were assembled into 24 chromosome-size scaffolds ranging from 11 Mb to 31 Mb in length and corresponding to the expected karyotype of trevally (Table 1). The remaining scaffolds (<0.8 % of the total assembly) that could not be anchored to pseudo-chromosomes were smaller, ranging from 1 kb to 51.2 kb in size.

**Table 1:** The 24 anchored trevally chromosomes following [45] and the corresponding scaffold names and their respective lengths (bp). Note, that the scaffold_001800 is located on Chromosome 21, making this the sex chromosome

Anchored chromosome	Scaffold name	Length (bp)
Chr01	scaffold_000114	24,151,137
Chr02	scaffold_000147	28,820,665
Chr03	scaffold_001154	27,945,907
Chr04	scaffold_001701	22,194,978
Chr05	scaffold_000836	22,952,508
Chr06	scaffold_001460	31,062,029
Chr07	scaffold_000312	24,419,205
Chr08	scaffold_000453	26,980,729
Chr09	scaffold_001891	28,251,306
Chr10	scaffold_001025	26,936,208
Chr11	scaffold_001200	18,865,379
Chr12	scaffold_000118	26,912,610
Chr13	scaffold_001584	22,008,664
Chr14	scaffold_000429	25,278,635
Chr15	scaffold_001123	23,296,617
Chr16	scaffold_000874	26,415,528
Chr17	scaffold_000314	22,912,052
Chr18	scaffold_001965	19,902,634
Chr19	scaffold_001072	19,893,011
Chr20	scaffold_000070	24,159,416
Chr21^a^	scaffold_001800	27,791,086
Chr22	scaffold_000657	25,934,954
Chr23	scaffold_000099	16,759,215
Chr24	scaffold_000328	11,005,663
Total anchored		574,850,136
Unanchored scaffolds		4,556,253
Total anchored + unanchored		579,406,389

^a^sex-linked

### Repeat and gene annotation

A total of 12.8 % of the genome was masked for repeats. BUSCO analysis of the anchored Trevally_v1 genome yielded a complete BUSCO score of 92.4 % with 2.4 % being single copy and 27.0 % being duplicated copies (134 were fragmented and 61 missing). In total, 28,416 protein-coding gene models were detected.

### Whole genome re-sequencing and detection of sex-determining regions

The number of paired reads from each of the 13 trevally F_0_ broodstock aligned to the reference genome ranged from 53,250,512 to 70,547,632 with a mean read number of 61,800,138. Read mapping rates ranged between 96.54 and 97.31 %, with between 90.00 % and 90.74 % of reads properly paired. With the majority of trimmed reads being 125 bp and with the reference genome size being 579,406,389 bp, the mean read depth for each fish was approximately 13X.

**Fig. 2:**
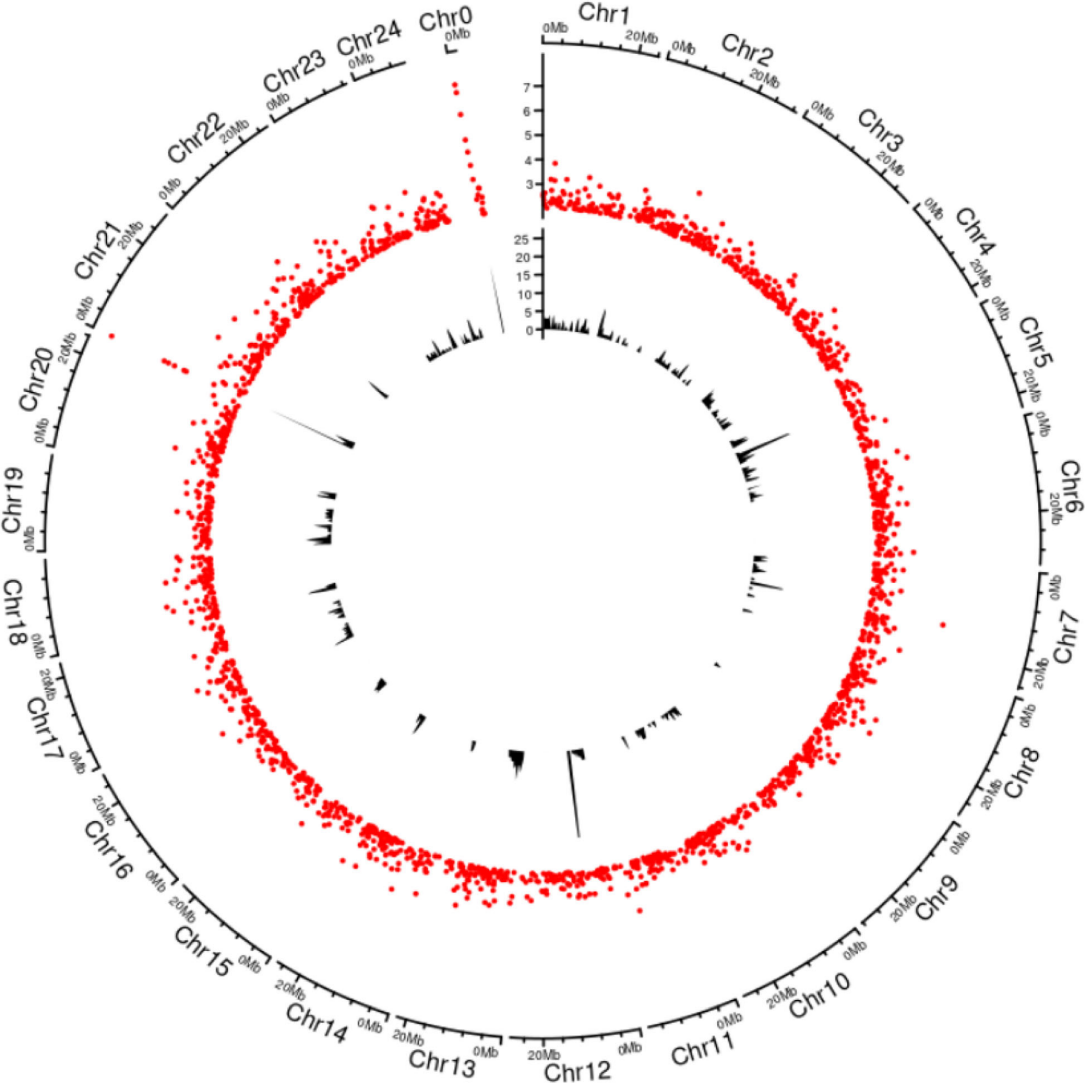
Genome-wide association of sex determining regions in the trevally genome. Circos plot of –log_10_ *P* values above 2, from t-tests of mean read depth variation within 1 kb windows, of whole genome sequence between male and female trevally (outer track in red), and of densities of variants whose state (heterozygous or homozygous) is fully sex-associated (inner track in black). Chr0 is composed of unanchored scaffolds. The regions of high densities of fully-associated variants that correspond to peaks of high –log_10_ P values of read-depth variation at the start of Chr21 and within Chr0 are from variants which are heterozygous in males and homozygous in females, while those of Chr5, Chr7 and Chr12 are from variants that are heterozygous in females and homozygous in males

In total 16,576,890 variants were detected, including 14,355,149 and 2,221,741 SNPs and indels, respectively. A total of 572 of these variants showed absolute association with sex, with all individuals of either sex genotyping as heterozygous while all others of the other sex phenotype as homozygous. Five pseudochromosomes contain regions of variants whose state is associated with sex at densities higher than 10 variants per 5 kb window: Chr5, Chr7, Chr12, Chr21 and Chr0 (Fig. 2). Associations in Chr0 involved three unanchored scaffolds; scaffold_000353, scaffold_000374 and scaffold_001951 of 5647 bp, 3469 and 4013 bp in size, respectively. Only those regions of Chr21 and Chr0 were associated with sex-associated read depth variation, specifically tracts of zero read depth in females.

**Fig. 3:**
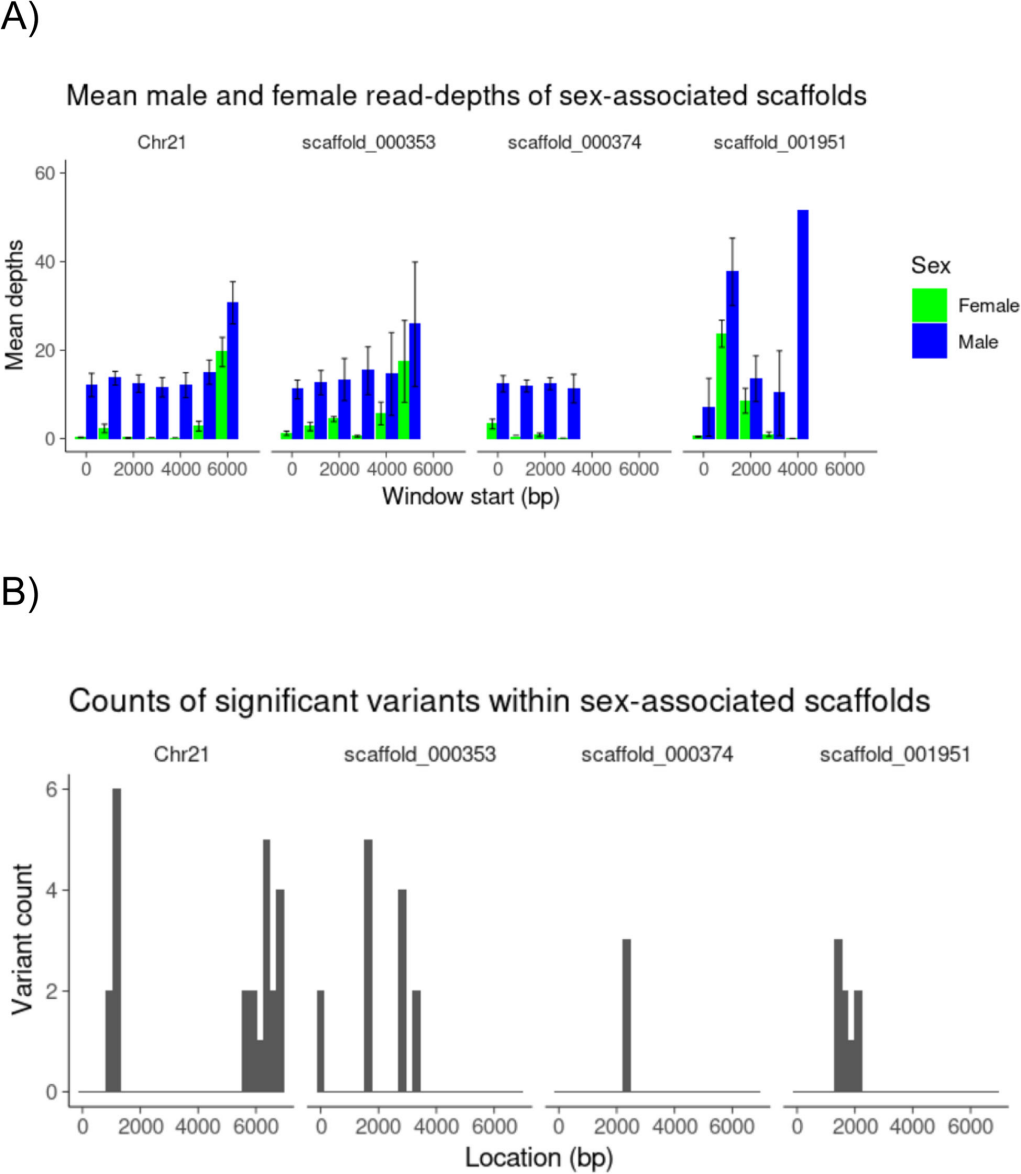
**A** Mean depths with standard deviations of aligned reads of males and females within 1 kb windows, along the first 6 kb of Chr21, and along the full lengths of scaffold_000353 (5647 bp), scaffold_000374 (3469 bp) and scaffold_001951 (4013 bp). The standard deviation of the mean read depth of males in scaffold_001951 (62.32) was removed for clarity. **B** A histogram of variants whose state is entirely associated with sex (-log 10 P-value of 3.234), within the first 6 kb of Chr21, and along the full lengths of scaffold_000353, scaffold_000374 and scaffold_001951. Bins are 200 bp

Close inspection of scaffold_001951 indicates that this scaffold contains a repetitive sequence that carries variants that are heterozygous in males and homozygous in females. However, while all females have zero read depth over major tracts of this scaffold, some males also showed a lack of read mapping over those same regions (Fig. 3). Contrastingly, scaffold_000353 shows major tracts of zero read depth in all females, while all males show consistent read mapping across the whole scaffold. Neither of these scaffolds show any sequence similarity to any known genes.

Chr5, Chr7 and Chr12 show short regions of association of variant state with sex across short regions within each chromosome. These regions include variants that are heterozygous in females and homozygous in males but further investigation into these regions suggested that these associations were spurious. Specifically, regions of 5.5 kb of Chr5 (8714673-8720250), 2.2 kb of Chr7 (13579401-13581609) and 6.7 kb of Chr12 (7693603-7700312) carry variants that are heterozygous in females and homozygous in males, signal that suggests a ZZ/ZW system. These regions were investigated further for sex-associated variation in co-localised read-depth and for sequence similarity to known sequences. No read-depth variation was seen across of these regions and no significant similarity was detected of sequence from the regions of Chr5 and Chr7 to any known gene sequence (blastx; NCBI; nr peptide database, Evalue = 0.001). The region of Chr12 showed sequence similarity to homologues of TELO2-interacting protein 1, a protein that is part of the TTT complex that is involved in DNA damage response signalling [46], but no reports of any involvement in sex-determination were found. Taken together, as these regions of signal are not supported by additional evidence of read-depth variation and sequence similarity to genes known to be associated with sex-determination, they are most parsimoniously explained as false discoveries.

### Model for sex determination

Twenty-five and three variants were heterozygous for all seven re-sequenced males and homozygous for all six females in Chr21 and scaffold_000374, respectively (Supplementary Table 1). Furthermore, both of these scaffolds showed major tracts where read mapping was absent, or very low, in all females (Fig. 3). These two features, where males carry additional genomic sequence, with co-located sequence divergence seen in the form of higher frequencies of heterozygous variants, are characteristic of a XX/XY sex determining system. While regions of Chr5 and Chr12 carry variants that are heterozygous in females and homozygous in males, suggesting a ZZ/ZW system, there is no co-located variation in read-depth. We therefore propose that a XX/XY model for sex determination is most likely in this species.

### Identification of a candidate gene related to sex determination

A literature review of sex determination in fish uncovered 32 research publications and from these, a total of 132 candidate SD genes were collated, of which 64 were unique (Supplementary Table 2). We used these 64 candidate genes as queries to search the trevally reference genome using blastn resulting in 2100 High Scoring Pairs (HSPs) with E-values less than 1e^−6^ (Table 2). Of these, 19 were detected in Chr21 and six were detected in scaffold_000374 with e-values ranging from 3e^−50^ to 7e^−8^. All HSPs of these scaffolds were with accessions described as c*yp19a1a*, cytochrome P450 aromatase, and *cyp19b* (Table 2).

**Table 2:** HSPs of sex-associated regions from blastn analysis (v2.2.25), querying the trevally reference assembly with candidate sex determination genes found from a literature search

Chromosome/scaffold	HSP start	HSP start	E value	NCBI Accession	Gene description
Chr21	412	241	6.00E-48	AY273211.1	cytochrome P450 aromatase
Chr21	412	251	5.00E-29	NM_001105093.2	*cyp19a1b*
Chr21	412	244	5.00E-29	XM_020703654.2	*cyp19a1b*
Chr21	412	274	6.00E-28	XM_020703653.2	*cyp19a1b*
Chr21	412	274	7.00E-28	XM_011475857.2	*cyp19a1b*
Chr21	412	359	7.00E-08	HQ449733.1	cyp19a1a
Chr21	631	524	7.00E-28	HQ449733.1	cyp19a1a
Chr21	631	524	2.00E-17	AY273211.1	cytochrome P450 aromatase
Chr21	1211	1125	7.00E-23	HQ449733.1	cyp19a1a
Chr21	1223	1125	1.00E-15	AY273211.1	cytochrome P450 aromatase
Chr21	1494	1333	7.00E-38	HQ449733.1	cyp19a1a
Chr21	1494	1342	1.00E-25	AY273211.1	cytochrome P450 aromatase
Chr21	1755	1610	4.00E-20	AY273211.1	cytochrome P450 aromatase
Chr21	1710	1606	3.00E-11	HQ449733.1	cyp19a1a
Chr21	2010	1857	1.00E-29	AY273211.1	cytochrome P450 aromatase
Chr21	1964	1869	1.00E-10	NM_001105093.2	*cyp19a1b*
Chr21	1964	1869	5.00E-09	XM_011475857.2	*cyp19a1b*
Chr21	1964	1869	5.00E-09	XM_020703653.2	*cyp19a1b*
Chr21	1964	1869	5.00E-09	XM_020703654.2	*cyp19a1b*
scaffold_000374	1810	1560	2.00E-37	AY273211.1	*cyp19a1b*
scaffold_000374	1804	1635	2.00E-23	NM_001105093.2	cytochrome P450 aromatase
scaffold_000374	1804	1635	8.00E-22	XM_020703653.2	cytochrome P450 aromatase
scaffold_000374	1804	1635	9.00E-22	XM_011475857.2	*cyp19a1b*
scaffold_000374	1804	1635	9.00E-22	XM_020703654.2	*cyp19a1b*
scaffold_000374	2359	2127	3.00E-50	AY273211.1	*cyp19a1b*

The highest hit from a blastn search using the first 3 kb of Chr21 (XP_018539124.1, predicted an aromatase of 521 amino acids from *Lates calcarifer*) was used to develop a best estimation of a gene model. In Chr21, the model consists of eight putative exons in the reverse orientation between 3037 and 244 bp, with similarity spanning from position 19 to position 353 of the 521 aa peptide of the query gene. No stop codons exist within the regions of similarity, which are often seen in pseudogenes. In scaffold_000374, the model consists of two exons between 2356 and 1539 bp with similarity that spans from position 354 to 521 of the query peptide. Likewise, no stop codons exist within the regions of similarity. The high level of sequence similarity along with the absence of premature stop codons leads us to propose that the gene is a functional aromatase of 531 amino acids within the predicted sex-associated regions, and that scaffold_000374 is part of the chromosome of pseudochromosome Chr21 and is likely to precede the pseudochromosome in sequence order. Coordinates of the exons of the gene model are shown in Supplementary Table 3.

**Fig. 4:**
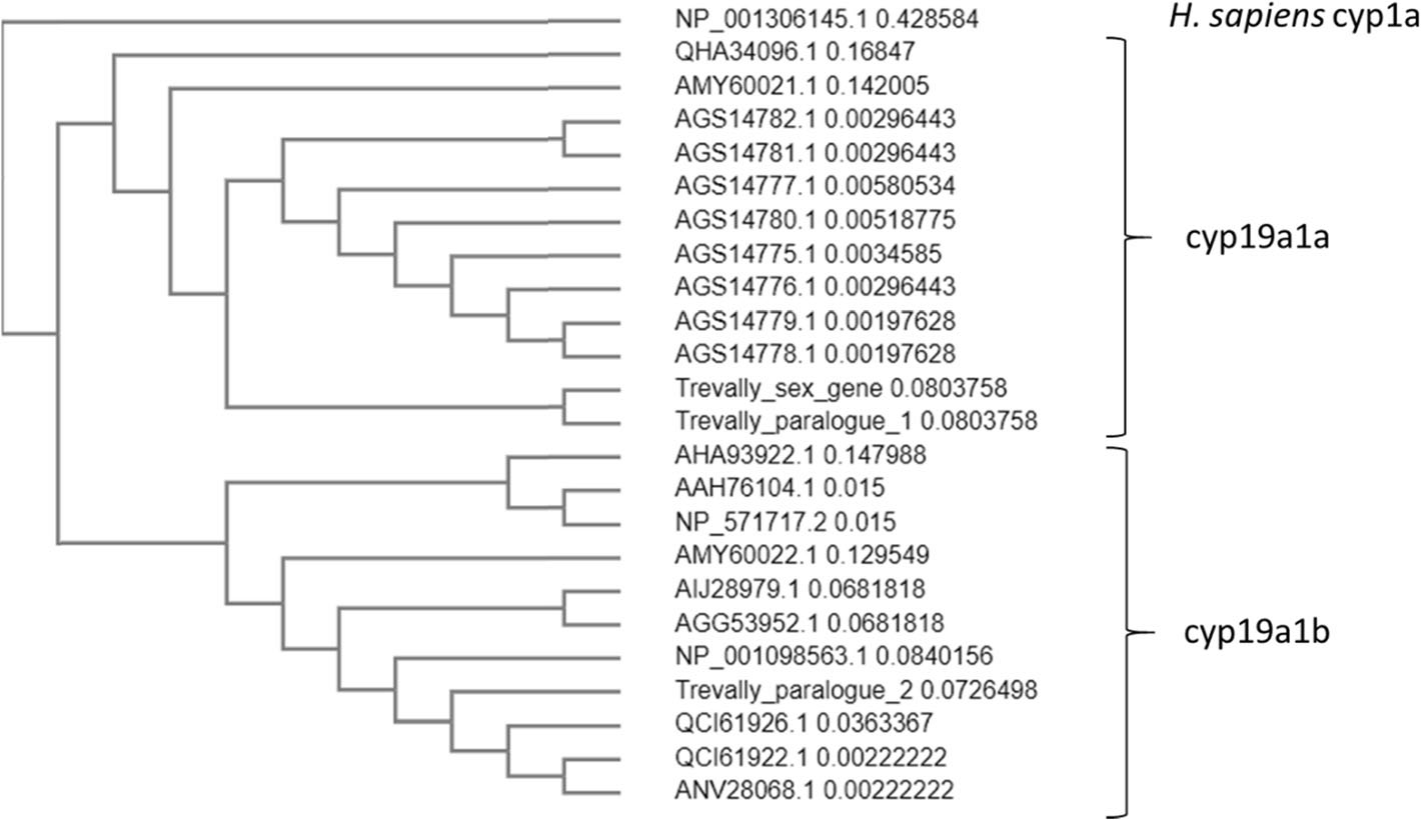
Guide tree based on peptide sequences of ten NCBI entries of each of *cyp19a1a* and *cyp19a1b*, along with the trevally sex-associated aromatase (Trevally_sex_gene) and two trevally paralogues (Trevally_paralogue_1 and Trevally_paralogue_2). A human *cyp1a* sequence was included as an outgroup

Models of two additional aromatase gene paralogues were similarly developed (see Supplementary Table 3). These consist of a proposed sequence of 479 aa, located on scaffold_001201 and scaffold_001906 (Trevally paralogue 1), and of 445 aa located on Chr16 (Trevally paralogue 2). On alignment with representative sequences of *cyp19a1a* and *cyp19a1b*, and construction of a guide tree, the proposed sex-associated aromatase and Trevally paralogue 1 clustered with sequences of *cyp19a1a*, while Trevally paralogue 2 clustered with sequences of *cyp19a1b* (Fig. 4). Taken together, these data indicate that the sex-associated aromatase is an additional paralogue of *cyp19a1a*.

### Sex marker development and validation

**Fig. 5:**
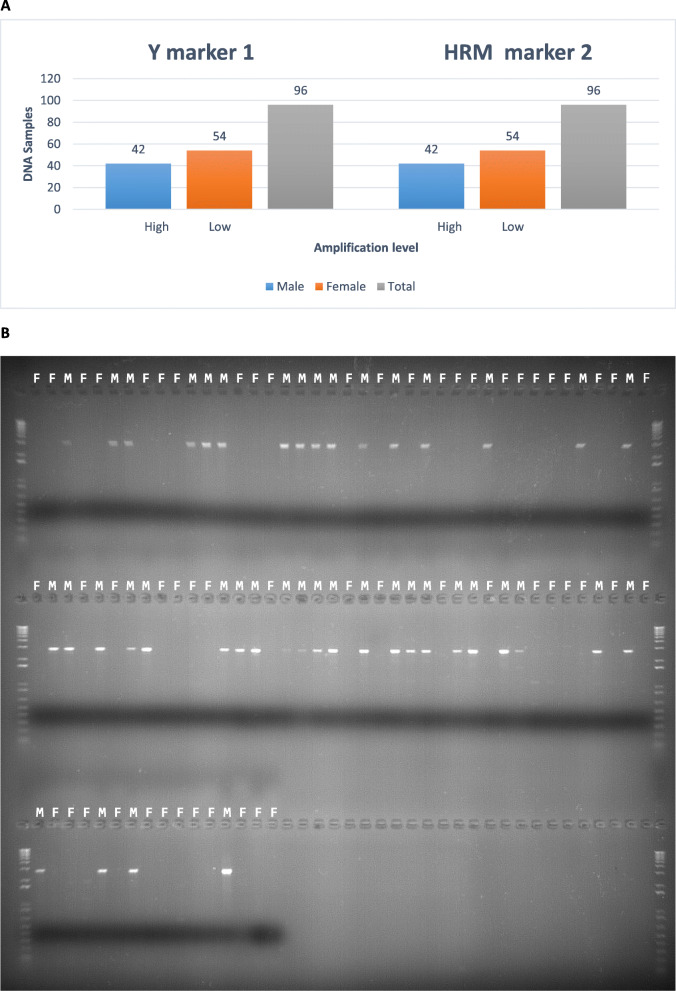
**A** Bar plot made from Y marker (‘FW1/RV1_412_241’) and HRM marker 2 (‘FW2/RV2_2359_2127’) measured cycle amplification levels from running HRM on a LightCyler480 (Roche) real time PCR instrument for males (blue) and females (orange). The Y-axis is the number of DNA samples and the labels high or low indicate amplification levels. **B** the full and uncropped 0.9 % agarose gel stained with RedSafe^TM^, which represents PCR products (~2.5 kb) from the sex-specific gene-wide marker (‘TRE_Cyp19a_FW1/RV1’). Text above each slot containing either an M (Male) or F (Female)

Three types of markers were designed and tested based on the candidate gene regions: Gene wide markers (*n*=5, Supplementary Table 4), Y-allele markers (*n*=15, Supplementary Table 5), and HRM markers (*n*=15, Supplementary Table 6), respectively. One (‘FW2/RV2_2359_2127’) out of the total 15 successfully amplified HRM markers (Supplementary Table 6) and one (FW1/RV1_412_421’) of the total 15 Y-allele specific designed markers (Supplementary Table 5) were linked to the sex trait and showed complete genotype to phenotype concordance on the 96 sexually characterized trevally (Fig. 5 A). Of the gene wide specific amplification markers (Supplementary Table 4) designed by amplifying large fragments of the *cyp19a* candidate gene, two (‘TRE_Cyp19a_FW1/RV1’ and ‘TRE_Cyp19a_FW2/RV2’) amplified a PCR product present in all males and absent from the females (Fig. 5B shows just the first marker’s results).

## Discussion

Here we present the first near-complete genome assembly of New Zealand trevally (*Pseudocaranx georgianus*) and one of the first for a carangid species, and the use of this and whole-genome variation of males and females to identify sex-linked regions. A genome assembly for golden pompano (*Trachinotus oyatus*) was assembled at the chromosome level [14]. Another genome for California yellowtail (*Seriola dorsalis*) was developed, however, it was not resolved into chromosome scale scaffolds [47]. Our assembly covers the 24 chromosomes expected from the family Carangidae; it is highly contiguous and only includes a small proportion of scaffolds that could not be anchored. The trevally genome will be useful to assemble other Carangidae fragmented genomes based on synteny, such as other *Seriola* spp. which are economically important for aquaculture around the globe [48]. The genomic resources developed in this work will also enable more routine genotyping work, for example, the genome will assist analyses that require linkage disequilibrium knowledge, SNP marker checking and selection, and Gene by Environment Association (GEA) analyses of wild trevally populations, to reveal local adaptation of different stocks. The sex marker will prove to be a valuable resource to determine sex in aquaculture breeding programmes, particularly in immature fish, to ensure optimal sex ratios in elite broodstock lines. The downside of a presence/absence assay is, however, that it does not include a control for PCR failure which can lead to males with failed PCRs being mis-classified as females. This can be prevented by adding an internal control, e.g. a second pair of primers to amplify a region of different length. The ability to genetically sex trevally will also facilitate to partition datasets into sex, and to determine sex-linked and impacted traits, to characterise sex specific trade-offs in growth and maturation, and to identify possible sex-linked physiological optima. We discuss our approach and findings and outline resulting applications and implications, and provide insights how our results improve the overall understanding of the genetics of sex determination in teleost fishes.

We chose two strategies to reveal the genomic regions linked to SD in trevally. First, we screened for genomic variants that were commonly, or always, in the heterozygous state in one sex and a homozygous state in the other. We discovered four regions with high numbers of variants seen in the heterozygous state in all seven male fish assessed, which were homozygous in all six female fish assessed. One region was approximately 6 kb and located at the proximal end of a chromosome-scale scaffold (Chromosome 21), while the other three regions span three short scaffolds (scaffold_000353, scaffold_000374 and scaffold_001951 of 5647 bp, 3469 and 4013 bp in size, respectively). The second strategy was based on read-depth variation between the sexes. We found higher read-depth in males compared to females along the same four scaffolds. Because the re-sequenced females showed absent regions compared to the reference Trevally_v1 assembly, we now hypothesise that the unsexed juvenile fish used as a specimen for genome assembly must have been a male. Our results also underscore the need for studies to go beyond SNPs in their data analysis and to include the wider spectrum of structural genomic variants, including copy number repeats such as insertions, duplication and deletions, as well as fusions, fissions and translocations, to increase the power of SD detection and to better detail the full extent of sexually divergent regions [49, 50]. An increasing number of studies, including on teleost species [51], reveal that structural genomic variants encompass more genome-wide bp variants compared to SNPs, and thus hold an enormous potential to act as a potent substrate in processes involved in the eco-evolutionary divergence of species.

The region linked to sex determination on the pseudo-chromosome scaffold_001800 (Chromosome 21) is small (~6 kb), and could have been easily missed with other methods involving less comprehensive variant detection, such as reduced-representation genotyping by sequencing (GBS). This illustrates how our strategy, using a full genome assembly coupled with the full re-sequencing of sexed individuals, efficiently enabled us to pinpoint this region, develop sex-specific markers, and identify a candidate gene. Interestingly, the sex-linked short scaffolds may be unanchored due to difficulties in resolving the genome assembly in the SD region. The divergence between the Y and X alleles may have prevented the Meraculous assembler from collapsing both haplotypes. Long read sequencing and a phased assembly would be useful to resolve this issue in the future.

In contrast to mammals and birds, cold-blooded vertebrates, and in particular teleost fishes, show a variety of strategies for sexual reproduction [52]. Sex chromosomes in teleosts can either be distinguishable cytologically (heteromorphic) or appear identical (homomorphic). In both cases, one sex is typically heterogametic (possessing two different sex chromosomes) and the other one homogametic (a genotype with two copies of the same sex chromosome). A male-heterogametic system is called an XX-XY system, and female-heterogametic systems are denoted as ZZ-ZW, and both types can be found side by side in closely related species [52]. Close relatives of trevally show the ZZ-ZW type of sex-determination; e.g. the Japanese amberjack (*Seriola quinqueradiata*). Evidence for a ZZ-ZW type of sex-determination would come from a higher number of heterozygous SNPs in females combined with a higher number of deletions in males (the latter would be hinting at a lack of the W-chromosome) [15]. In another closely related species, golden pompano, a SNP found within the gene Hsd17b1 was detected as a sex-specific marker, being heterozygous in females and homozygous in males, also supporting a ZZ-ZW system in this species. Yet in trevally the opposite is seen. When examining the SD region between the sexes, we found that in all instances males were heterozygous while all females were homozygous. A similar pattern was seen in the number of deletions. Of the 418 deletions detected, all of these were located in females, whereas none were located in males, when comparing the data with our male reference genome (note that male-specific deletions would only be observed if our reference genome was that of XX female due to Y degradation). Taken together, this all strongly indicates that trevally has XX-XY sex determining system.

Other teleost fish with a similar XX-XY sex determining mechanism have been well described. In the Atlantic cod (*Gadus morhua*), studies found deletions in females (hinting lack of a Y-chromosome) and males showed high SNP heterozygosity on the sex determination gene zKY [53] (confirmed with diagnostic PCR). In Medaka (*Oryzias latipes*) the XX-XY sex chromosomes were determined using genetic crosses and the tracking of sex linked markers [54]. Recent studies have also revealed the putative sex gene for two carangids, the greater amberjacks (*Seriola dumerili*) and Californian yellowtails (*Seriola dorsalis*), [15]. Biochemical analyses in greater amberjacks showed a missense SNP in the Z-linked allele of 17β-hydroxysteroid dehydrogenase 1 gene (Hsd17b1) [55]. In Californian yellowtails, Hsd17b1 was found in the SDR, identified by deletions in the female sex, like the SDR in trevally, however, females and not males were heterogametic in yellowtails [47]. The Hsd17b1 gene catalyses the interconversion of estrogens (estrone<->estradiol) and androgens (androstenedione<-> and testosterone). The Hsd17b1 gene can thus be classified as an estradiol-synthesising sex determination gene, just like cyp19 [15], because cyp19 converts androgens to estradiol (testosterone->estradiol).

Our results provide strong evidence that two small genomic regions form the major part of the SD locus of trevally. The presence of a *Cyp19a1a*-like gene within these sex-associated regions, strongly implicates a role of this gene in the sex determination of this species. No reads from female fish aligned to the gene sequence and male-specific PCR amplification of markers based on the gene indicate that it is specific to male fish and suggest it might play a role in the masculinisation of genetically male fish.

Previous research has demonstrated that *cyp19a1b* catalyses the irreversible conversion of the androgens androstenedione and testosterone into the estrogens estrone and estradiol, respectively [56]. Recent genomic investigations have detailed that the two variants of the *cyp19 gene* (*cyp19a1a* and *cyp19a1b*) seen in most teleost fish were derived from the teleost specific whole genome duplication (3R) and evolved through sub-functionalisation [57]. Expression of variant A (*cyp19a1a*) is restricted to the gonads (mainly the ovary), whereas the B variant (*cyp19a1b*) is expressed in the brain and the pituitary [58]. When looking at studies of the genus *Seriola*, which is in the same family as trevally, variant A is only expressed in the ovaries [59]. For males, the presence of this gene appears to be related to spermatogenesis and testicular development in some species [60], something that is also found in other vertebrate species outside of teleosts [61]. Stage-specific gene expression during spermatogenesis in European bass (*Dicentrarchus labrax)* gonads, for example, has revealed that *cyp19a1a* at lower levels has a regulatory effect at the initial stages of spermatogenesis [55]. In addition to this regulatory effect, *cyp19a1a* has also been implicated in the differentiation of sex in black porgy (*Acanthopagrus schlegeli)*, where high levels were expressed during early testicular development [62]. Females still have higher expression than males of this gene at any ontogenetic stages, however. This is probably because as well as regulation and differentiation of the ovary at higher levels during early sex differentiation [63], for females this gene is also an important factor in the female reproductive cycle [64].

Variant B, which is expressed mostly in the brain, is attributed to the control of reproduction and behaviour related to sex. RT-PCR analysis of the hermaphroditic mangrove killifish (*Rivulus marmoratus)* showed that *cyp19a1b* is expressed in both the male and hermaphroditic fish, whilst *cyp19a1a* was completely absent in males [65]. In addition, a study where *cyp19a1b* levels were artificially lowered in male guppy (*Poecilia reticulate)* showed these fish experience a reduction in the performance of male specific behaviours [66]. Females also express *cyp19a1b*, but this expression is mainly restricted to the period around spawning. Work on both zebrafish (*Danio rerio*) and channel catfish (*Ictalurus punctatus*) show an increase in *cyp19a1b* right before the onset and during spawning, while a decrease and low levels *cyp19a1b* are found outside of the reproductive period [58]. Taken together, these studies are consistent with a *cyp19a1b* being more male-linked compared to *cyp19a1a*, and conversely, that *cyp19a1a* is associated with female phenotypes.

The presence of a sex-associated *cyp19a1a-*like gene, in addition to autosomal paralogues of *cyp19a1a* and *cyp19a1b*, implies a duplication of *cyp19a1a* and up-recruitment to assume the role of the sex determining gene in trevally. Up-recruitment of key SD genes from lower in the sex-determination pathway was originally proposed by Wilkins [67] and a number of examples supporting the hypothesis are seen among teleost fish. In *Oryzias latipes*, sex determination is mediated in the embryo by *Dmy*, a Y-specific duplication of *Dmrt1*, which itself is expressed much later during development of the testes [68]. Other similar examples include *GsdfY* in *Oryzias luzonensis* [69] and *amhy* in *Hypoatherina tsurugae* [70]. Evidence of the suitability of *cyp19a1a* for duplication and up-recruitment as an SD master gene is seen in the plasticity of the gene, shown by its common recruitment among cichlids into roles other than its usual role of aromatisation of androgens into estrogens within the ovaries [71]. Further research is required to elucidate what the role of *cyp19a1a*-like is, and better understand its function in sex determination of trevally. Final determination of up-recruitment of *cyp19a1a* to the sex-associated aromatase as the master SD gene of trevally, will require closer analysis of expression of the sex-associated aromatase, *cyp19a1a* and *cyp19a1b* within the brain, testes and ovaries during development.

## Conclusions

As a greater number of fish genomes are sequenced, it is likely that more genes involved in the regulation of sex will be discovered. This will provide much needed data for future comparative genomic work to track the evolutionary processes and patterns governing sex evolution across close and distant teleost lineages. Given the importance of trevally and other carangid species for aquaculture production (e.g. *Seriola*) and wild fisheries [18], our reference genome will contribute to accelerating marker-assisted breeding programmes, and will aid genomics-informed fisheries management programmes, by providing insights into sex ratios and sex specific effects [72]. This genome assembly was a key resource to detect a sex-determining region in trevally, and it will be a substantial resource for a variety of research applications such as population genomics and functional genomics, in both cultured and wild populations of this and other carangid species. The developed resources will further studies into teleost evolution, specifically the evolution of sex determination, which has proven to be a complex and highly variable trait in fish.

## Supplementary information

**Additional file 1.**

## Data Availability

The genome sequence data are considered taonga (i.e. a treasured resource to the indigenous people of Aotearoa New Zealand, the Māori) and therefore data are hosted in an Aotearoa New Zealand-based data repository. This was done to recognise Māori as important partners in science and innovation and as inter-generational guardians of significant natural resources and indigenous knowledge. Further studies using this material, raw sequencing data and final genome assembly will require consent from the Māori iwi (tribe) who exercises guardianship for this material according to Aotearoa New Zealand’s Treaty of Waitangi and the international Nagoya protocol on the rights of indigenous peoples. Raw and analyzed data are available through the Genomics Aotearoa data repository at https://repo.data.nesi.org.nz/. Contact Maren Wellenreuther to discuss access (maren.wellenreuther@plantandfood.co.nz). The genomic information described here will be made available to researchers provided relevant kaitiaki agree with this, and it can be demonstrated that Māori core cultural values will be respected and the intended use of the data will have a positive impact on Māori social, cultural, environmental and economic well-being.
