# Supplementary material for: The genome of New Zealand trevally (Carangidae: Pseudocaranx georgianus) uncovers a XY sex determination locus
Source: BMC Genomics. 2021 Nov 2;22:785. doi: 10.1186/s12864-021-08102-2 (PMC8561880; doi:10.1186/s12864-021-08102-2)
Supplement: Supplementary file 1 — Additional file 1. [file 12864_2021_8102_MOESM1_ESM.docx]

Supplementary data

### **Supplementary Material 1. Fish rearing details**

F_0_ broodstock (n=21; mean body weight 1.4 ± 0.5 kg; mean fork length 44.3 ± 5.6 cm) were captured from two trawl fishing tows from the North Taranaki Bight (Lat. 3845267- Long. 17420626 and Lat. 3851887- Long. 17419780) during February 2012. Captured fish were transported to the Wakefield Quay Finfish Facility (formerly operated by PFR in Nelson, New Zealand) and acclimated to a single 4400 L tank. Broodstock (remaining n=19) were later transferred to the Maitai Finfish Facility (currently operated by PFR in Nelson, New Zealand) in 2014 and were acclimated to a single 13000 L tank.

Spawning was induced to produce the F_1_ population in December 2015 when the ambient water temperature had reached 17.9°C. In brief, the tank was fitted with an external passive egg collector and broodstock (mean body weight 3.5 ± 0.9 kg; mean fork length 51.4 ± 3.7 cm) received an intraperitoneal injection of human chorionic gonadotropin (Chorulon®) at a target dose of 600 IU/ kg bodyweight. Following injection, two individuals died at three and five days post-injection. The egg collector was checked daily (between 8 am and 9 am) for spawned eggs. Egg release was first observed at 48 h post-injection, and at approximately 7–9 days post-injection, 50 g of buoyant eggs were collected for incubation over three consecutive days (fertilisation rates and egg production metrics were unreported). Eggs were transferred to individual 450 L upwelling conical incubators supplied with gentle aeration and ambient seawater (temp range: 19.4–21.7°C). At hatching, F_1_ larvae were combined into a single 5,000L tank where they were fed enriched rotifers *(Brachionus plicatilis)*, newly hatched artemia nauplii (*Artemia franciscana*) and reared using a semi-static green water rearing protocol. When larvae reached a notochord length of ~7 mm, they were weaned onto fully enriched *Artemia salina* followed by dry crumb (O.range, INVE Aquaculture) and an in-house wet diet consisting of minced seafood. At approximately 77 days post-hatching, all fish were transferred into a single 63,000 L tank for on-growing under the husbandry conditions described above.

### **Supplementary Material 2. RNA extraction for transcriptome sequencing**

Skin, white muscle, gill, liver, kidney, brain and heart tissues were collected from five randomly selected F_1_ offspring and immediately placed in RNAlater. RNA was extracted per tissue (five replicates/tissue) with the CTAB buffer described above as follows: approximately 50 mg of tissue were processed as for the DNA preps until the aqueous phase was obtained after the second CIA extraction. At this point the aqueous phase was precipitated with 0.35 volumes of 8 M LiCl, mixed by inversion and incubated at 4°C overnight. The RNA was collected by centrifugation at 16,000 *g* in a refrigerated micro centrifuge. The pellet was dissolved in 500 µL SSTE buffer (1 M NaCl, 0.5% SDS, 1 mM EDTA, 10 mm Tris-HCl pH 8.0) and extracted once with an equal volume of CIA. The aqueous phase was collected after 10 min centrifugation at 16,000 *g* and precipitated with 2 volumes of 100% ethanol. The RNA was collected by centrifugation at 16,000 *g* and washed with 1 mL of 70% ethanol, then air dried at room temperature for approximately 30 min. The RNA was dissolved in 200 µL sterile deionised water. The RNA quality was assessed by absorbance ratios (260/280 nm and 260/230 nm) and was quantified by absorbance at 260 nm. The samples were DNase-treated with the Ambion® TURBO DNA-free™ kit (Thermo-Fisher Scientific, Waltham, MS, USA) as directed by the manufacturer. The RNA quality and quantity were assessed by absorbance, as described above. The fragment-size distribution was assessed by capillary electrophoresis using a Fragment Analyzer (Advanced Analytical, Parkersburg, WV, USA), using the High Sensitivity RNA Analysis kit.

The RNA samples were sequenced at the Australian Genome Research Facility (AGRF). The best replicate from each tissue was selected for the transcriptome dataset by preparing TrueSeq libraries (Illumina, San Diego, CA, USA). The rest of the samples were prepared with the Lexogen QuantSeq 3’mRNA kit (Lexogen, Wien, Austria) for the expression analysis dataset.

### **Supplementary Material 3. DNA extraction**

**DNA extraction for the genome sequencing**

Heart tissue was collected from a two-year-old F_1_ individual and immediately preserved in RNAlater (Sigma-Aldrich, St Louis, USA) as recommended by the manufacturer. Total genomic DNA was extracted from a subsample (~20 mg) placed in 750 µL of CTAB buffer (2% hexadecyltrimethyl ammonium bromide, 2% polyvinyl pyrrolidinone K40, 2 M NaCl, 25 mM EDTA, 100 mM Tris-HCl pH 8.0) containing 2% β-mercaptoethanol. The tissue was disrupted by hand with a sterile plastic pestle until a homogeneous mixture was obtained. The homogenised samples were then extracted twice by vortexing with one volume of chloroform:isoamyl alcohol (CIA, 24:1) for 10 sec to remove the denatured proteins and centrifuged at 16,000 g for 5 min to separate solid and aqueous phases. The final aqueous phase was then transferred to a new 1.5 mL screw-capped tube and the genomic DNA was precipitated by adding 0.7 volumes of room temperature isopropanol and left to precipitate at -20°C for at least 1 h. The DNA was collected by centrifugation at room temperature for 10 min at 16,000 g. The DNA pellet was washed with 1 mL of 70% ethanol (v/v). After all traces of ethanol were removed by air drying, DNA was slowly dissolved in 100 µL sterile TE buffer (10 mM Tris-HCl pH 7.5, 1 mM EDTA) at 4°C overnight. RNA was removed by adding 4 µL of RNase A (100 mg/µL) to the DNA and incubating for 5 min at room temperature. Following RNase treatment, samples were subjected to a final CTAB extraction as described above. The final DNA pellet was dissolved in 100 µL of TE buffer and quantified by fluorescence (Qubit™ HS dsDNA kit Invitrogen). DNA quality was assessed by standard gel electrophoresis (1% agarose in 1X TAE buffer, 40 mM Tris-acetate, 1 mM EDTA at pH 8.3) and by pulse field gel electrophoresis in 1% Certified™ Megabase Agarose (Bio-Rad) in 1X TAE buffer. The average fragment size of the DNA was 40 kbp.

**DNA extraction of the broodstock (Whole genome sequencing WGS)**

DNA was extracted as follows: approximately 20 mg of finclip tissue were added to a mixture of 400 µL extraction buffer (0.4 M NaCl, 10 mM Tris-HCl pH 8.0, and 2 mM EDTA pH 8.0) and 80 µL of 10% SDS. The sample was incubated at 80°C for 5 min and immediately cooled on ice. Ten microliters of Proteinase K (10 mg/mL) were added and mixed by inversion. The sample was incubated at 56°C for 1.5 h. The insoluble material was removed by centrifugation at 16,000 *g* for 5 min. The supernatant was transferred to a new micro centrifuge tube and the proteins were salted out by adding 320 µL 5 M NaCl and mixing by inversion. The denatured proteins were collected by centrifugation at 16,000 *g* for 5 min. The clear supernatant was transferred to a new micro centrifuge tube and the RNA was removed by adding 5 µL RNase A 100 µg/µL, incubated at room temperature for 5 min. The sample was centrifuged at 16,000 *g* for 5 min and the supernatant was transferred to a new micro centrifuge tube. The DNA was precipitated with 525 µL isopropanol and incubated at -20°C overnight, prior to collection by centrifugation at 16,000 *g* for 10 min. The pellet was washed with 1 mL of 70% ethanol, dried and resuspended in 100 µL TE buffer. DNA quality was assessed by absorbance ratios (A_260/280_ nm and A_260/230_ nm) and the quantified by fluorescence (Qubit™ HS dsDNA kit, Invitrogen). The integrity of the DNA was assessed by capillary electrophoresis using the High Sensitivity Genomic DNA Analysis kit on the Fragment Analyzer (Illumina).


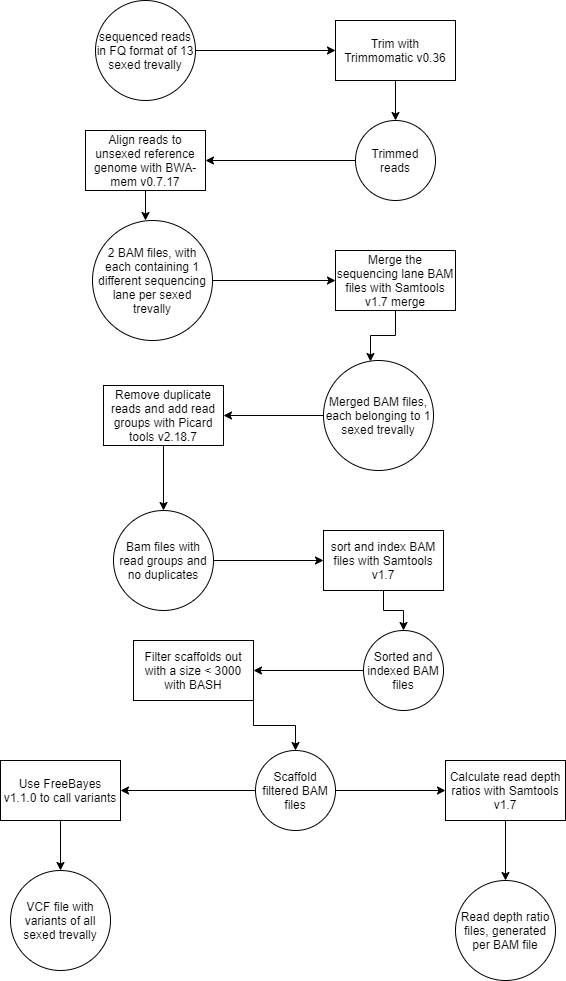


### **Supplementary Fig. 1.** The overview of generating read depth and variant data from the sequences genomes of 13 sexed trevally.

| **Sex** |  | **Male** | **Female** | **Male** | **Female** | **Male** | **Female** | **Female** | **Female** | **Male** | **Female** | **Male** | **Male** | **Male** |
| --- | --- | --- | --- | --- | --- | --- | --- | --- | --- | --- | --- | --- | --- | --- |
| **Assembly scaffold** | **Position (bp)** | **F0_01** | **F0_02** | **F0_03** | **F0_04** | **F0_05** | **F0_06** | **F0_07** | **F0_08** | **F0_09** | **F0_10** | **F0_11** | **F0_12** | **F0_13** |
|  |  |  |  |  |  |  |  |  |  |  |  |  |  |  |
| scaffold001800 (Chr21) | 1033 | G\|T | T\|T | G\|T | T\|T | G\|T | T\|T | T\|T | T\|T | G\|T | T\|T | G\|T | G\|T | G\|T |
| scaffold001800 (Chr21) | 1041 | T\|A | A\|A | T\|A | A\|A | T\|A | A\|A | A\|A | A\|A | T\|A | A\|A | T\|A | T\|A | T\|A |
| scaffold001800 (Chr21) | 1080 | A\|T | T\|T | A\|T | T\|T | A\|T | T\|T | T\|T | T\|T | A\|T | T\|T | A\|T | A\|T | A\|T |
| scaffold001800 (Chr21) | 1083 | G\|A | A\|A | G\|A | A\|A | G\|A | A\|A | A\|A | A\|A | G\|A | A\|A | G\|A | G\|A | G\|A |
| scaffold001800 (Chr21) | 1084 | A\|G | G\|G | A\|G | G\|G | A\|G | G\|G | G\|G | G\|G | A\|G | G\|G | A\|G | A\|G | A\|G |
| scaffold001800 (Chr21) | 1086 | A\|G | G\|G | A\|G | G\|G | A\|G | G\|G | G\|G | G\|G | A\|G | G\|G | A\|G | A\|G | A\|G |
| scaffold001800 (Chr21) | 1163 | A\|G | G\|G | A\|G | G\|G | A\|G | G\|G | G\|G | G\|G | A\|G | G\|G | A\|G | A\|G | A\|G |
| scaffold001800 (Chr21) | 1164 | A\|G | G\|G | A\|G | G\|G | A\|G | G\|G | G\|G | G\|G | A\|G | G\|G | A\|G | A\|G | A\|G |
| scaffold000374 | 2297 | A\|G | G\|G | A\|G | G\|G | A\|G | G\|G | G\|G | G\|G | A\|G | G\|G | A\|G | A\|G | A\|G |
| scaffold000374 | 2350 | A\|G | G\|G | A\|G | G\|G | A\|G | G\|G | G\|G | G\|G | A\|G | G\|G | A\|G | A\|G | A\|G |
| scaffold000374 | 2396 | A\|G | G\|G | A\|G | G\|G | A\|G | G\|G | G\|G | G\|G | A\|G | G\|G | A\|G | A\|G | A\|G |

### **Supplementary Table 1.** Overview of variants in female and male re-sequenced trevally in assembly scaffolds linked to the SDR.

### **Supplementary Table 2.** Description of the 64 sex candidate genes mined from literature [40, 57, 62-88]. All candidate genes are sorted alphabetically on the gene sex region description column.

| **Candidate sex gene description** |
| --- |
| *adcy5 (adenylate cyclase 5),* patterning of fish colour ornaments in male fish. |
| *amh* (*anti-Müllerian hormone*), male sex differentiation in mammals, regulation of sex hormones, dimorphic in brain development. |
| *amhr2* (*anti-Müllerian hormone (AMH) receptor type 2),* involved in male sex differentiation. |
| *amhy (anti-Müllerian hormone on Y-chromosome),* a duplicated version of *amh* that found its way onto the Y chromosome. |
| *ar (androgen receptor),* a nuclear receptor that is activated by androgenic hormone binding. |
| *bdrt (bromodomain protein transcript variant 2),* see below. |
| *brdt (bromodomain testis associated,)* involvement in elongating spermatids, and is required for proper spermatogenesis and male fertility. |
| *copz-1 (coatomer protein complex, subunit zeta-1),* development of colour dimorphism between sexes. |
| *csf1ra (colony stimulating factor 1 receptor a),* type III receptor tyrosine kinase of egg spot tissue related to sex. |
| *ctnnb1* (*catenin (cadherin-associated) beta*)*,* intracellular signal transducer, part of the wntpathway, interactions with androgens, teleost A-copy. |
| *cyp11b2 (cytochrome P450 family 11 subfamily B polypeptide 2)*, steroid hydroxylase, contributes to the synthesis of 11-ketotestosterone, the most potent androgen in teleosts. |
| *cyp17a1 (family 17, subfamily A, polypeptide 1),* formation (synthesis) of steroid hormones. This group of hormones includes sex hormones such as testosterone and estrogen. |
| *cyp19a1a* (*aromatase cytochrome P450 family 19 subfamily A polypeptide 1 A*), monooxygenase involved in estrogen biosynthesis, teleost A-copy (also known as ovarian aromatase). |
| cyp19a1b (aromatase cytochrome P450 family 19 subfamily A polypeptide 1), see above, teleost Bcopy (also known as brain aromatase). |
| *dax1 (dosage-sensitive sex reversal adrenal hypoplasia critical region on chromosome X gene 1)*, orphan receptor, negative regulates steroidogenesis, interacts with *sf-1*. |
| *dhh (desert hedgehog),* cell signalling involved in male sex differentiation (in the form of morphogenesis). |
| *dmo (DM-domain gene in ovary),* DM-domain cDNA in ovary. |
| *dmrt1 (doublesex and mab-3 related transcription factor 1*), transcription factor, regulation of male sex differentiation. |
| *dmrt1bY (doublesex and mab-3 related transcription factor 1b on Y-chromosome),* a duplicated version of *Dmrt1* that found its way onto the Y chromosome. |
| *dmrt3 (doublesex and mab-3 related transcription factor 3*), involved in male sex determination. |
| *esr1 (estrogen receptor 1)*, receptor that is activated by the estrogen hormone. |
| *fgf20 (fibroblast growth factor 20)*, growth factor involved in male sex development. |
| *fgf9 (fibroblast growth factor 9)*, growth factor involved in male sex development. |
| *fhl2* (*the four and a half LIM domain protein 2*), Egg spot formation related to sex. |
| *Follistatin (fst),* ovarian differentiation (through follicle stimulating hormone). |
| *foxl2* (*forkhead transcription factor L2*), transcription factor, possible role in ovarian development and function. |
| *gata4 (GATA binding protein 4)*, transcription factor, reproductive/gonad development and function. |
| *GM354, UNH168, GM271, UNH131, UNH971, UNH213, UNH868,* Presumed sex determination region based on male/female marker testing. |
| *gsdf (gonadal somatic cell derived factor)*, gonadal soma derived growth factor on the Y chromosome. |
| *hsd17b1 (hydroxysteroid 17-beta dehydrogenase 1), a dual function in estrogen activation and androgen inactivation.* |
| *hsd17b3 (hydroxysteroid 17-beta dehydrogenase 3 protein coding)*, catalyses the conversion of androstenedione to testosterone. |
| *kiss2r (Kiss2 receptor)*, role in reproductive function in vertebrates, particularly in the onset of puberty. |
| *kita (receptor tyrosine kinases),* required for the development of colour ornaments of males. |
| *mc4r (melanocortin 4 receptor)*, male size and behaviour polymorphisms. |
| *nanos1A (Nanos homolog 1 A),* brain and germ cell development, teleost A-copy. |
| *nanos1B (Nanos homolog 1 B),* see above, teleost B-copy. |
| *nr5a1a* (*nuclear receptor subfamily 5, group A, member 1 A*), an important factor for steroidogenesis and sex differentiation. |
| *nr5a2 (nuclear receptor subfamily 5, group A, member 2)*, involved in steroidogenesis, also known as lrh-1 or FTZ-F1. |
| *nr5a5* (*nuclear receptor subfamily 5, group A, member 5*), related to *sf-1,* gene of the *nr5a* family, regulation of sterol/steroid metabolism. |
| *pax7 (paired box 7 in PAX family of transcription factors),* regulation of melanocytes related to sex. |
| *pdgfa (platelet derived growth factor subunit A),* signalling molecules involved in male sex differentiation. |
| *pdgfrb (platelet derived growth factor receptor beta),* signalling molecules involved in male sex differentiation. |
| *rspo1* (*R-spondin-1*), activates wnt/beta-catenin pathway in ovarian differentiation in mammals. |
| *sdy (the sexually dimorphic on the Y-chromosome gene),* a conserved male-specific Y-chromosome sequence. |
| *sequ21, Sequ320, Sequ230 (marker regions),* sexual determining marker regions based on linkage analysis. |
| *Sf-1* (*nr5a1, steroidogenic factor 1*), orphan nuclear receptor, controls sexual development and Reproduction. |
| *shbg (sex hormone-binding globulin),* globulin that binds sex hormones on its receptor. |
| *shp (short heterodimer partner, NR0B2)*, development Ligand-independent activation(due to homology classified as a receptor) of *ESR1* and *ESR2*. |
| slc45a2 (*solute carrier family 45 member 2*), part of sex linkage group required for sex related chromatophores. |
| *sox10* (*SRY (sex determining region Y)-box 10*), regulating male sexual development. |
| sox3 (*SRY (sex determining region Y)-box 3*), necessary for initiating male sex determination by directing the development of supporting cell precursors. |
| *sox8* (*SRY (sex determining region Y)-box 8*), regulating male sexual development. |
| *sox9* (*SRY (sex determining region Y)-box 9*), transcription factor, regulates *amh* expression together with *sf-1*, gonad development. |
| Spra (*Sepiapterin Reductase*), part of sex linkage group required for sex related chromatophores. |
| *srd5a1 (steroid 5 alpha-reductase 1),* catalyses the conversion of testosterone into the more potent androgen, dihydrotestosterone. |
| *srd5a2 (steroid 5 alpha-reductase 2),* catalyses the conversion of testosterone into the more potent androgen, dihydrotestosterone. |
| *srd5a3 (steroid 5 alpha-reductase 3),* catalyses the conversion of testosterone into the more potent androgen, dihydrotestosterone.22 |
| *Star (steroidogenic acute regulatory protein),* cholesterol transference in mitochondria. |
| *tbx1a (T-box transcription factor 1 a),* t‐box transcription factor gene family involved in embryogenesis and organogenesis, teleost A-copy. |
| *tbx1b (T-box transcription factor 1 b),* see above, teleost B-copy. |
| *tdrd1* (*Tudor domain containing 1*), presumed germ cell formation. |
| *wnt4* (*wingless-type MMTV integration site family member 4*), presumed to be crucial for mammalian ovary development. |
| *wt1A* (*Wilms tumour 1 A*), transcription factor, gonad development, interacts with *sf-1.* |
| *xmrk (receptor tyrosine kinase)*, pigment pattern formation related to sex. |

### **Supplementary Table 3.** Bed coordinates the proposed model of the sex-associated gene of trevally and the two paralogues. The gene models were developed based on the peptide sequence most similar to the first 3 kb of Chr21 based on a blastx query of the NCBI nr Protein database.

| Sex associated cyp19a1a-like |  |  |  |  |  |
| --- | --- | --- | --- | --- | --- |
|  | Exon1 | scaffold_001800 | 2821 | 3037 | minus |
|  | Exon2 | scaffold_001800 | 1859 | 2006 | minus |
|  | Exon3 | scaffold_001800 | 1599 | 1755 | minus |
|  | Exon4 | scaffold_001800 | 1325 | 1493 | minus |
|  | Exon5 | scaffold_001800 | 1100 | 1217 | minus |
|  | Exon6 | scaffold_001800 | 523 | 631 | minus |
|  | Exon7 | scaffold_001800 | 244 | 412 | minus |
|  | Exon8 | scaffold_000374 | 2110 | 2356 | minus |
|  | Exon9 | scaffold_000374 | 1538 | 1808 | minus |
|  |  |  |  |  |  |
| Trevally Paralogue 1 |  |  |  |  |  |
|  | Exon1 | scaffold_001906 | 1820 | 1880 | minus |
|  | Exon2 | scaffold_001906 | 1664 | 1799 | minus |
|  | Exon3 | scaffold_001906 | 621 | 771 | minus |
|  | Exon4 | scaffold_001906 | 358 | 514 | minus |
|  | Exon5 | scaffold_001906 | 79 | 247 | minus |
|  | Exon6 | scaffold_001201 | 2288 | 2396 | minus |
|  | Exon7 | scaffold_001201 | 2004 | 2172 | minus |
|  | Exon8 | scaffold_001201 | 916 | 1156 | minus |
|  | Exon9 | scaffold_001201 | 351 | 603 | minus |
|  |  |  |  |  |  |
| Trevally Paralogue 2 |  |  |  |  |  |
|  | Exon1 | scaffold_000874 | 17147575 | 17147734 | plus |
|  | Exon2 | scaffold_000874 | 17147829 | 17147982 | plus |
|  | Exon3 | scaffold_000874 | 17148098 | 17148176 | plus |
|  | Exon4 | scaffold_000874 | 17148188 | 17148275 | plus |
|  | Exon5 | scaffold_000874 | 17148402 | 17148519 | plus |
|  | Exon6 | scaffold_000874 | 17148606 | 17148720 | plus |
|  | Exon7 | scaffold_000874 | 17148810 | 17148978 | plus |
|  | Exon8 | scaffold_000874 | 17148996 | 17149020 | plus |
|  | Exon9 | scaffold_000874 | 17149089 | 17149302 | plus |
|  | Exon10 | scaffold_000874 | 17149389 | 17149611 | plus |

### **Supplementary Table 4.** Primers names, sequence information, expected product sizes and annealing temperatures, for validation PCR. Accession numbers of orthologous genes at NCBI of the gene-wide designed primers are given. The cells with bold letters represent the primers that were successful.

| **Gene wide primers** | | | | | | |
| --- | --- | --- | --- | --- | --- | --- |
| **Name of the primer** | **Sequence** | **Location** | **Start_end** | **Size product** | **Annealing temperature** | **Gene origin** |
| **TRE_Cyp19a_FW1** | **GGAAGTCGTGCATGTTCAAAG** | **trevally001800** | **92_112** | **649** | **60** | **HQ449733.1** |
| **TRE_Cyp19a_RV1** | **TGGTCTGTCTGCTGCTGGT** | **trevally001906** | **1717_1699** | **649** | **60** | **HQ449733.1** |
| **TRE_Cyp19a_FW2** | **TGAAAGGAAGTCGTGCATGTT** | **trevally001800** | **87_107** | **654** | **60** | **HQ449733.1** |
| **TRE_Cyp19a_RV2** | **TGGTCTGTCTGCTGCTGGT** | **trevally001906** | **1717_1699** | **654** | **60** | **HQ449733.1** |
| TRE_Cyp19a_FW3 | CGTGCATGTTCAAAGTCAAATC | trevally001800 | 98_119 | 643 | 60 | HQ449733.1 |
| TRE_Cyp19a_RV3 | TGGTCTGTCTGCTGCTGGT | trevally001906 | 1717_1699 | 643 | 60 | HQ449733.1 |
| TRE_Cyp19a_FW4 | ATACTGCATTTCCGGTTCCA | trevally000314 | 7881589_7881575 | 610 | 60 | HQ449733.1 |
| TRE_Cyp19a_RV4 | TGGTCTGTCTGCTGCTGGT | trevally001906 | 1717_1699 | 610 | 60 | HQ449733.1 |
| TRE_Cyp19a_FW5 | TACTGCATTTCCGGTTCCA | trevally000314 | 7881589_7881575 | 609 | 60 | HQ449733.1 |
| TRE_Cyp19a_RV5 | TGGTCTGTCTGCTGCTGGT | trevally001906 | 1717_1699 | 609 | 60 | HQ449733.1 |

### **Supplementary Table 5.** Primers names, sequence information, expected product sizes and annealing temperatures for PCR testing. The gene origin accession codes of the Y-only (Y-chromosome parts). The cells with bold letters represent the primers that were successful.

| **Y-only primers** | | | | |
| --- | --- | --- | --- | --- |
| Primer name | Sequence | Size product | Annealing temperature | Gene origin |
| FW1_631_524 | TCAGTGGTGAAGTTGATGTGGTC | 111 | 60.74 | HQ449733.1 |
| RV1_631_524 | TGTGTTGTGCTTCAGTCAGGAG | 111 | 60.74 | HQ449733.1 |
| FW2_631_524 | TGTGGTCAAGCTTCTCCGC | 98 | 60.30 | HQ449733.1 |
| RV2_631_524 | GTGTGTGTTGTGCTTCAGTCAG | 98 | 60.22 | HQ449733.1 |
| FW3_631_524 | GTGAAGTTGATGTGGTCCAGCT | 103 | 60.81 | HQ449733.1 |
| RV3_631_524 | TGTTGTGCTTCAGTCAGGAGC | 103 | 60.81 | HQ449733.1 |
| FW4_631_524 | GTTGATGTGGTCCAGCTTCTCC | 102 | 61.20 | HQ449733.1 |
| RV4_631_524 | TGTGTGTTGTGCTTCAGTCAGG | 102 | 61.26 | HQ449733.1 |
| FW1_1494_1333 | AGACTGTGCAGCTGGTCCA | 76 | 61.14 | HQ449733.1 |
| RV1_1494_1333 | CAGGTCCAGGTCTGCAGAAC | 76 | 60.32 | HQ449733.1 |
| FW2_1494_1333 | TGTCCTCTCACCGTCCACAG | 113 | 61.18 | HQ449733.1 |
| RV2_1494_1333 | TGGACCAGCTGCACAGTCT | 113 | 61.14 | HQ449733.1 |
| **FW1_412_241** | **GGTGTCGATCTCCTCCAGCA** | **104** | **61.32** | **NM_001105093.2 &**  **AY273211.1** |
| **RV1_412_241** | **TGATCGCGGCTCCAGTAACT** | **104** | **61.32** | **NM_001105093.2 &**  **AY273211.1** |
| FW2_412_241 | GTCGATCTCCTCCAGCAGCT | 79 | 61.39 | NM_001105093.2 &  AY273211.1 |
| RV2_412_241 | GTCCATCAGCCTCTTCTTCATGC | 79 | 61.54 | NM_001105093.2 &  AY273211.1 |
| FW1_1810_1560 | CAGGTTGTTGGTCTGCAGGA | 83 | 60.18 | NM_001105093.2 &  AY273211.1 |
| RV1_1810_1560 | TCCTGGTGACGCTGCTTTC | 83 | 60.30 | NM_001105093.2 &  AY273211.1 |
| FW2_1810_1560 | GTCGGCTCAGGAAGGTCATG | 84 | 60.46 | NM_001105093.2 &  AY273211.1 |
| RV2_1810_1560 | AGACCAACAACCTGTGGCAG | 84 | 60.47 | NM_001105093.2 &  AY273211.1 |
| FW3_1810_1560 | CAGTTTCCTCTATGTCGGCTCAG | 100 | 60.74 | NM_001105093.2 &  AY273211.1 |
| RV3_1810_1560 | TGCAGACCAACAACCTGTGG | 100 | 60.75 | NM_001105093.2 &  AY273211.1 |
| FW4_1810_1560 | TTCCAGTTTCCTCTATGTCGGC | 106 | 60.09 | NM_001105093.2 &  AY273211.1 |
| RV4_1810_1560 | TCCTGCAGACCAACAACCTG | 106 | 60.18 | NM_001105093.2 &  AY273211.1 |
| FW1_2010_1857 | AGCGTCTCCTCTCCGTTGATC | 117 | 61.62 | AY273211.1 |
| RV1_2010_1857 | GGGGCCTCTTCTCTCCTACATG | 117 | 61.61 | AY273211.1 |
| FW2_2010_1857 | ATCCAAACTCTCACCATGTCTCC | 100 | 60.05 | AY273211.1 |
| RV2_2010_1857 | TGGGGCCTCTTCTCTCCTAC | 100 | 60.03 | AY273211.1 |

### **Supplementary Table 6.** Primer names, sequences, size of product, annealing temperature for PCR and gene origin accession code of the HRM designed primers for validation. The cells with bold letters represent the primers that turned out to be successful markers.

| **HRM primers** | | | | | | |
| --- | --- | --- | --- | --- | --- | --- |
| **Name primer** | **Sequence** | **Location** | **Start_end** | **Size product** | **Annealing temperature** | **Gene origin** |
| FW1_2359_2127 | GATGATGTTTGTGCCTCTTGGG | trevally000374 | 2195_2216 | 195 | 59.83 | AY273211.1 |
| RV1_2359_2127 | ACTGACAGGTTGGTTCCTCT | trevally000374 | 2389_2370 | 195 | 58.19 | AY273211.1 |
| **FW2_2359_2127** | **GGATGATGTTTGTGCCTCTTGG** | **trevally000374** | **2194_2215** | **196** | **59.83** | **AY273211.1** |
| **RV2_2359_2127** | **ACTGACAGGTTGGTTCCTCT** | **trevally000374** | **2389_2370** | **196** | **58.19** | **AY273211.1** |
| FW3_2359_2127 | TGAGGATGATGTTTGTGCCTCT | trevally000374 | 2191_2212 | 199 | 59.69 | AY273211.1 |
| RV3_2359_2127 | ACTGACAGGTTGGTTCCTCT | trevally000374 | 2389_2371 | 199 | 58.19 | AY273211.1 |
| FW4_2359_2127 | GATGATGTTTGTGCCTCTTGGG | trevally000374 | 2195_2216 | 195 | 59.83 | AY273211.1 |
| RV4_2359_2127 | ACTGACAGGTTGGTTCCTCTTC | trevally000374 | 2389_2371 | 195 | 59.9 | AY273211.1 |
| FW5_2359_2127 | GGATGATGTTTGTGCCTCTTGG | trevally000374 | 2194_2215 | 196 | 59.83 | AY273211.1 |
| RV5_2359_2127 | ACTGACAGGTTGGTTCCTCTTC | trevally000374 | 2389_2371 | 196 | 59.9 | AY273211.1 |
| FW1_1211_1125 | TGCTGAAATGGCTCCTACAACA | trevally001800 | 1049_1070 | 195 | 60.22 | HQ449733.1 |
| RV1_1211_1125 | GTGTGTGAACTGTGTGTGTGT | trevally001935 | 2533_2551 | 195 | 59.2 | HQ449733.1 |
| FW2_1211_1125 | TGCTGAAATGGCTCCTACAAC | trevally001800 | 1049_1069 | 195 | 58.56 | HQ449733.1 |
| RV2_1211_1125 | GTGTGTGAACTGTGTGTGTGT | trevally001935 | 2533_2551 | 195 | 59.2 | HQ449733.1 |
| FW3_1211_1125 | TGCTGAAATGGCTCCTACAACA | trevally001800 | 1049_1070 | 196 | 60.22 | HQ449733.1 |
| RV3_1211_1125 | AGTGTGTGAACTGTGTGTGTG | trevally001800 | 1244_1226 | 196 | 58.92 | HQ449733.1 |
| FW4_1211_1125 | TGCTGAAATGGCTCCTACAACA | trevally001800 | 1049_1070 | 197 | 60.22 | HQ449733.1 |
| RV4_1211_1125 | GAGTGTGTGAACTGTGTGTGTG | trevally001800 | 1245_1227 | 197 | 59.91 | HQ449733.1 |
| FW5_1211_1125 | GCTGAAATGGCTCCTACAACA | trevally001800 | 1050_1070 | 195 | 58.56 | HQ449733.1 |
| RV5_1211_1125 | AGTGTGTGAACTGTGTGTGTG | trevally001800 | 1244_1226 | 195 | 58.92 | HQ449733.1 |
| FW1_13047900_13048000 | CAAGGGTTGGGTCTTCTCGG | trevally001025 | 13047916_13047935 | 100 | 60.32 | No gene |
| RV1_13047900_13048000 | CCAGGCTGCTTCATCCTCTT | trevally001025 | 13048015_13047996 | 100 | 59.74 | No gene |
| FW2_13047900_13048000 | ACAAGGGTTGGGTCTTCTCG | trevally001025 | 13047915_13047933 | 101 | 59.6 | No gene |
| RV2_13047900_13048000 | CCAGGCTGCTTCATCCTCTT | trevally001025 | 13048015_13047996 | 101 | 59.74 | No gene |
| FW3_13047900_13048000 | TTCTCGGACCCTGGACTGTA | trevally001025 | 13047929_13047948 | 87 | 59.59 | No gene |
| RV3_13047900_13048000 | CCAGGCTGCTTCATCCTCTT | trevally001025 | 13048015_13047996 | 87 | 59.74 | No gene |
| FW4_13047900_13048000 | AAGGGTTGGGTCTTCTCGGA | trevally001025 | 13047917_13047936 | 99 | 60.47 | No gene |
| RV4_13047900_13048000 | CCAGGCTGCTTCATCCTCTT | trevally001025 | 13048015_13047996 | 99 | 59.74 | No gene |
| FW5_13047900_13048000 | CAAGGGTTGGGTCTTCTCGG | trevally001025 | 13047916_13047935 | 102 | 60.32 | No gene |
| RV5_13047900_13048000 | GTCCAGGCTGCTTCATCCTC | trevally001025 | 1304801713047999 | 102 | 60.46 | No gene |
